# N^6^-Methyladenosine-Modified circRNA in the Bovine Mammary Epithelial Cells Injured by *Staphylococcus aureus* and *Escherichia coli*

**DOI:** 10.3389/fimmu.2022.873330

**Published:** 2022-04-04

**Authors:** Haojun Xu, Changjie Lin, Ting Li, Yifan Zhu, Jinghan Yang, Sijie Chen, Jianguo Chen, Xi Chen, Yingyu Chen, Aizhen Guo, Changmin Hu

**Affiliations:** ^1^ Department of Clinical Veterinary Medicine, College of Veterinary Medicine, Huazhong Agricultural University, Wuhan, China; ^2^ State Key Laboratory of Agricultural Microbiology, Huazhong Agricultural University, Wuhan, China; ^3^ The Center for Animal Disease Control and Prevention in Wuhan, Wuhan Bureau of Agriculture and Rural Bureau Affairs, Wuhan, China; ^4^ Department of Preventive Veterinary Medicine, College of Veterinary Medicine, Huazhong Agricultural University, Wuhan, China

**Keywords:** N^6^-methyladenosine, circRNA, epithelial cells, cell injury, inflammation, *S. aureus*, *E. coli*

## Abstract

Mastitis is a common disease that hinders the development of dairy industry and animal husbandry. It leads to the abuse of antibiotics and the emergence of super drug-resistant bacteria, and poses a great threat to human food health and safety. *Staphylococcus aureus* (*S. aureus*) and *Escherichia coli* (*E. coli*) are the most common pathogens of mastitis in dairy cows and usually cause subclinical or clinical mastitis. CircRNAs and N^6^-methyladenosine (m^6^A) play important roles in immunological diseases. However, the mechanisms by which m^6^A modifies circRNA in bovine mammary epithelial cells remain poorly understood. The aim of our study was to investigate m^6^A-modified circRNAs in bovine mammary epithelial cells (MAC-T cells) injured by *S. aureus* and *E. coli.* The profile of m^6^A-modified circRNA showed a total of 1,599 m^6^A peaks within 1,035 circRNAs in the control group, 35 peaks within 32 circRNAs in the *S. aureus* group, and 1,016 peaks within 728 circRNAs in the *E. coli* group. Compared with the control group, 67 peaks within 63 circRNAs were significantly different in the *S. aureus* group, and 192 peaks within 137 circRNAs were significantly different in the *E. coli* group. Furthermore, we found the source genes of these differentially m^6^A-modified circRNAs in the *S. aureus* and *E. coli* groups with similar functions according to GO and KEGG analyses, which were mainly associated with cell injury, such as inflammation, apoptosis, and autophagy. CircRNA–miRNA–mRNA interaction networks predicted the potential circRNA regulation mechanism in *S. aureus-* and *E. coli*-induced cell injury. We found that the mRNAs in the networks, such as BCL2, MIF, and TNFAIP8L2, greatly participated in the MAPK, WNT, and inflammation pathways. This is the first report on m^6^A-modified circRNA regulation of cells under *S. aureus* and *E. coli* treatment, and sheds new light on potential mechanisms and targets from the perspective of epigenetic modification in mastitis and other inflammatory diseases.

## Introduction

Mastitis is currently the most widely investigated disease in dairy cows and is usually caused by infection with specific pathogenic microorganisms (1). Currently, treatment with antibiotic causes the abuse of antibiotics and the emergence of super drug-resistant bacteria. It is a great threat to human health. For instance, methicillin-resistant *Staphylococcus aureus* (MRSA) from bovines is now the most serious acute infectious disease in hospital, neonatal delivery room, and community (2). *S. aureus* and *Escherichia coli* are the most common pathogens that cause mastitis, and the infection has variable clinical symptoms ranging from different pathogens (3). The characteristics of subclinical mastitis caused by *S. aureus* are chronic or invisible, which makes disease detection and therapy difficult (4). Conversely, mastitis caused by *E. coli* and some Gram-negative bacteria usually passes through acutely and is associated with a list of severe symptoms (5). Bovine mammary epithelial cells, as the first layer, have an essential defense function, and the tight junctions of the cells provide a barrier to enhance natural mammary immunity. The cells secrete inflammatory factors to fight bacterial infections when activated (6). Moreover, it will express a set of cytokine/chemokine factors and directly resist pathogens, such as bactericidal β-defensins (7), complement factors, and acute phase proteins (8, 9). This response occurs through an inflammatory-related pathway (10–13). Some studies have confirmed the immune response of mastitis in *S. aureus* and *E. coli* infection (14–18). However, the regulatory function of noncoding RNA and epigenetic modification is ignored.

Circular RNA (circRNA) has attracted considerable critical attention in recent years. As an indispensable noncoding RNA, circRNA is constituted by a highly stable and conserved covalent closed loop structure without a 3’ cap and 5’ polyA structure in organisms. Depending on the source gene of circRNA, different studies reported that the circRNA could be separated into different categories (19–21), such as exonic circRNA, intergenic circRNA, intronic circRNA, antisense circRNA, and sense overlapping circRNA. CircRNAs have been reported to bind to miRNAs as sponges and regulate target gene expression by combining with miRNAs (22). With the development of sequencing technology, many researchers have found that circRNAs widely exist in immune cells (23–25). Additionally, KEGG and GO analyses indicated that circRNAs played an essential role in many pathways associated with inflammation and immunity, such as the NF-κB, NLR, and TNF pathways (26). Therefore, circRNA has been considered a breakthrough in the innovative treatment of inflammatory disease.

M^6^A is a common epitranscriptome modification of RNA that is widely distributed in mammary epithelial cells (27). m^6^A has been found in diverse ribonucleic acids (28–31). By regulating RNA splicing (32), degradation (33), stability (34), and translation (35), the final manifestation is the impact on various pathological or physiological processes, such as the cell cycle (36) and immune function (37). Interestingly, circRNA could be regulated by m^6^A modification, showing a different m^6^A pattern from mRNA (38). Recent studies have shown that m^6^A-modified circRNAs have a certain impact on immune liver metastasis (39). Nevertheless, there is no study of the relationship between m^6^A-modified circRNAs and mastitis in dairy bovines.

To identify the potential function of m^6^A modification in regulating circRNA, we used RNA immunoprecipitation and high-throughput sequencing (MeRIP-seq) for the first time to profile the circRNA regulated by m^6^A modification in mammary epithelial cells (MAC-T) injured by inactivated *S. aureus* and *E. coli.* The features of m^6^A-modified circRNAs were identified first, and the potential functions were also predicted by GO enrichment analysis and KEGG pathway analysis. Moreover, a circRNA–miRNA–mRNA interaction network was constructed to predict competitive endogenous RNAs (ceRNAs). For further investigation, conjoint analysis of circRNA-seq and MeRIP-seq was performed. Together, our findings demonstrated the potential molecular function of circRNAs regulated by m^6^A modification caused by *S. aureus-* and *E. coli-*induced cell injury, which suggested that *S. aureus* and *E. coli* may affect the cell inflammation process through m^6^A-modified circRNAs, and provided us with possible diagnosis and treatment targets in mastitis.

## Materials and Methods

### Cell Lines and Bacteria

MAC-T cells (an immortalized bovine mammary epithelial cell line) were donated by Professor Mark Hanigan of Virginia Tech University. MAC-T cells were cultured by adapting the procedure used by Li et al. (40).

*S. aureus* (ATCC 29213) and *E. coli* (ATCC 25922) were donated by Professor Zhou Rui and Professor Wang Xiangru of Huazhong Agricultural University. *S. aureus* and *E. coli* were resuscitated, single colony purified, cultured, and heat-inactivated by adapting the procedure used by Li et al. (40).

### MAC-T Cell Injury Induced by Inactivated *S. aureus* and *E. coli*

MAC-T cells were seeded in 6-well plates (Corning, US) at a density of 2×10^5^ cells/well. After 12 h of culture, inactivated bacteria were added at an MOI of 10:1. Inactivated bacteria and cells were cocultured for 24 h. Cells in each well were washed 3 times with cold PBS (HyClone, China) before TRIzol (Invitrogen, US) was added. There were 3 replicate wells for each treatment and 3 replicate wells for the control, for a total of 9 samples.

### Extraction of Total RNA and RT-qPCR

Total RNA of the above 9 samples was extracted according to the instructions of the commercial reagent manufacturer and the RNA isolation steps. A NanoDrop ND-1000 instrument (Agilent Inc. USA) was used to measure RNA concentration and purity, and the quality control index of RNA purity was based on the OD260/OD280 value between 1.8 and 2.0. Denaturing agarose gel electrophoresis was used to measure RNA integrity and gDNA contamination. The sample was stored at −80°C for later use.

The RNA samples obtained were reverse transcribed into cDNA using Vazyme HiScript II Reverse Transcriptase (+gDNA wiper) (Vazyme, Nanjing, China), and the expression of related RNA was detected by Vazyme AceQ^®^ SYBR qPCR Master Mix (Vazyme) in a ViiA7 Real-time PCR System (Applied Biosystems Inc., Foster City, CA, United States). The 2^−△△ct^ method was used to analyze the fluorescence quantitative data, and GraphPad Prism 7.0 was used to process the data. Related primer sequences are shown at **Table S1**.

### Flow Cytometry

After MAC-T cells were digested, collected, and washed three times with PBS, the cells were resuspended in 100 µl of Binding Buffer. Then, 5 µl each of FITC and PI dyes (Vazyme, China) was added and left for 10 min at room temperature in the dark. After adding 400 µl of Binding Buffer, the cells were detected using CytoFLEX-LX (Beckman Coulter, Indianapolis, IN, USA).

### Transmission Electron Microscopy

MAC-T cells were treated with *S. aureus* and *E. coli* and fixed with 2% glutaraldehyde fixation buffer. The cells were used for TEM slide preparation. Sections were observed *via* a 100 KV H7650 transmission electron microscope (HITACHI, Japan).

### Establishment of circRNA Library

Nine samples under different treatments were sent to Cloud-Seq Biotech (Shanghai, China) for MeRIP-CircRNA sequencing. The operation was briefly described as follows: (1) To enrich circRNA, a circRNA Enrichment Kit (Cloud-seq, USA) was used according to the supplier’s instructions. (2) An NEBNext^®^ Ultra™ II Directional RNA Library Prep Kit (New England Biolabs, Inc., Massachusetts, USA) was used to pretreat RNA, and a sequencing library was constructed by immunoprecipitation. (3) Quality control and quantification of the library were performed with the BioAnalyzer 2100 system (Agilent Technologies, USA), and the Illumina HiSeq instrument was used to perform 150 bp paired-end sequencing.

### CircRNA Raw Data

After paired-end sequencing, raw circRNA data were obtained. According to Q30 for quality control, cutadapt software (v1.9.3) was used to obtain high-quality reads. STAR software (v2.5.1b) was used to compare high-quality reads to the reference genome/transcriptome (bosTau9), and DCC software (v0.4.4) was used for circRNA detection and identification. The circBase database and Circ2Traits were used to annotate the identified circRNA. Then, edgeR software (v3.16.5) was used for data standardization and differential expression circRNA screening (fold change ≥ 1.5, *p*-value ≤ 0.05). The standardized number of reads was used to calculate the differential expression of circRNA between the three groups of samples. A fold change ≥ 2.0 and *p*-value ≤ 0.05 were considered the thresholds for differential circRNA.

### Bioinformatics Analysis and Statistical Analysis

According to the circRNA verified by sequencing, the differentially expressed circRNA was selected. Kyoto Encyclopedia of Genes and Genomes (KEGG; http://www.genome.jp/keg, accessed on 27 April 2020) and Gene Ontology (GO; http://www.geneontology.org, accessed on 27 April 2020) enrichment analyses for the circRNA source gene were performed using the DAVID biometric analysis tool. The downstream mRNAs were predicted using miRBase (http://mirbase.org/index.shtml).

Statistical analysis of the data was performed using GraphPad Prism 7.0 (GraphPad Software, La Jolla, CA, United States). Data are represented as the mean with SD, and the significant differences were analyzed by Student’s *t*-test; *p* < 0.05 was considered statistically significant.

Additionally, RNAhybrid (2.1.2) was used to predict circRNA and miRNA interaction (41). Mirbase 22 (https://mirbase.org/, accessed on March 3, 2018) was used to predict the downstream mRNA of the predicted miRNA.

## Results

### Inflammation, Apoptosis, and Autophagy in MAC-T Cells Treated With Inactivated *S. aureus* and *E. coli*

**Figure 1:**
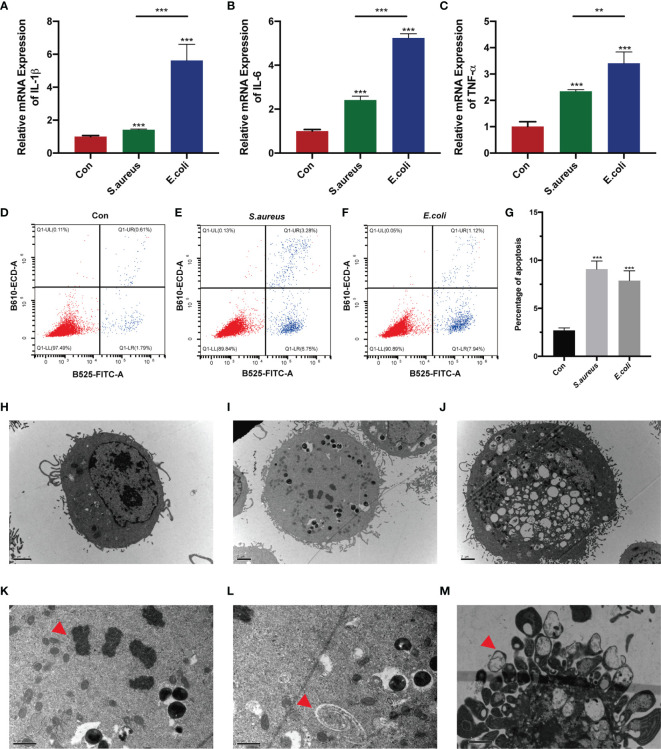
MAC-T cells injured by *S. aureus* and *E. coli*. **(A–C)** MAC-T cells were stimulated by inactivated *S. aureus* and *E. coli* and significantly higher IL-1β, IL-6, and TNF-α mRNA expression was found in the treatment groups. **(D–F)** Flow cytometry detected apoptosis in the *S. aureus* group and *E. coli* group. **(G)** The percentage of apoptotic cells in the *S. aureus* group and *E. coli* group was significantly higher than that in the control group. **(H–M)** Transmission electron microscopy (TEM) imaging of MAC-T cells. TEM illustrated **(H)** normal MAC-T cells, **(I)** MAC-T cells injured by *S. aureus*, and **(J)** MAC-T cells injured by *E. coli*. **(K)** Nuclear membrane disappearance, chromatin condensation (red arrow) and **(L)** autophagosomes (red arrow) were found in the *S. aureus* group. **(M)** Apoptotic bodies (red arrow) were found in the *E. coli* group (**p < 0.01, ***p < 0.001).

To determine whether the stimulation of inactivated bacteria on MAC-T cells can cause cell damage, we added bacteria to stimulate the cells for 24 h. The results showed that IL-1β, IL-6, and TNF-α were significantly upregulated in the *S. aureus* group and *E. coli* group, and the expression of these three genes in the *E. coli* group was significantly higher than that in the *S. aureus* group (**Figures 1A–C**). Additionally, cell apoptosis was significantly increased (**Figures 1D–F**). It showed almost 5 times the apoptosis rate in the *S. aureus* group and *E. coli* group compared to the control group. In contrast to the trend in mRNA expression associated with inflammation, flow cytometry illustrated a similar apoptosis rate when cells were treated with *S. aureus* and *E. coli* (**Figure 1G**).

To more intuitively observe the cell injury in *S. aureus* treatment and *E. coli* treatment, transmission electron microscopy was used (**Figures 1H–M**). Interestingly, we found the nuclear membrane disappearance, chromatin condensation (**Figure 1K**), and autophagosomes (**Figure 1L**) in MAC-T cells injured by *S. aureus* (**Figure 1I**), which indicated minor damage in these cells. However, cells with acute cytosolic voids (**Figure 1J**) and apoptotic bodies (**Figure 1M**) were found in MAC-T cells injured by *E. coli*.

### Identification of m^6^A-Modified circRNAs in MAC-T Cells Treated With Inactivated *S. aureus* and *E. coli*

To investigate the circRNA profile in MAC-T cells injured by heat-inactivated *S. aureus* or *E. coli*, purified cellular RNA was subjected to circRNA-seq and MeRIP-seq. The sequencing raw reads were generated from the control group, *S. aureus* group, and *E. coli* group. Bioinformatic approaches, including Cutadapt and Bowtie2 software, were performed for data filtering and quality control. We obtained clean reads and the proportions of net reads were between 99.122% and 99.995% with stringent quality control (**Table S2**).

To clarify the relationship between m^6^A modification and normal circRNA, we first analyzed the circRNA profile from circRNA-seq and found that most circRNAs were less than 2,000 bp and derived from exons (**Figures S1A, B**). Moreover, genes were mainly distributed on chromosome 3 (**Figure S1E**). When MAC-T cells were injured by *S. aureus* and *E. coli*, the source and length of the differential circRNA showed similar patterns (**Figures S1C, D, F**). Most circRNAs were located on chromosome 3 in the *S. aureus* group, while most circRNAs were located on chromosome 8 in the *E. coli* group (**Figures S1F, G**).

**Figure 2:**
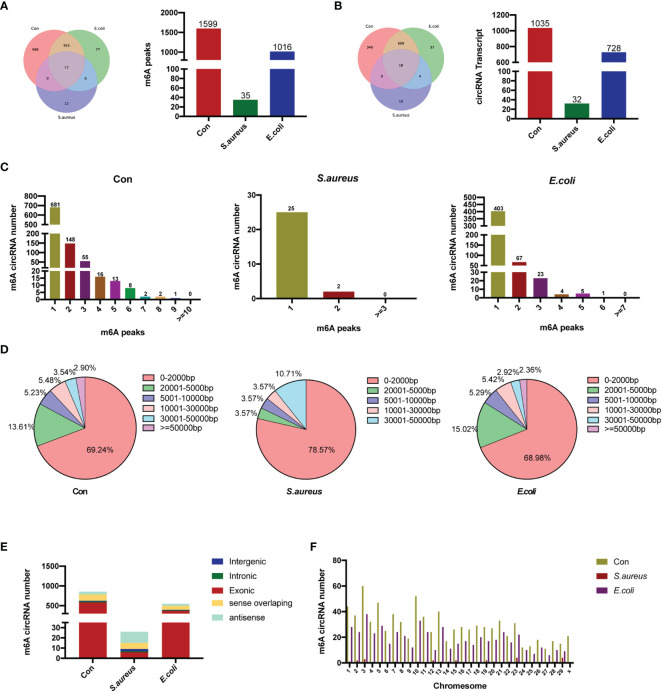
Overview of m^6^A-modified circRNAs in MAC-T cells injured by *S. aureus* and *E. coli*. **(A)** Venn diagram and histogram displaying the specific m^6^A peaks between the control group, *S. aureus* group, and *E. coli* group. **(B)** Venn diagram and histogram showing the specific circRNA with m^6^A modification between the three groups. **(C)** The number of m^6^A peaks per circRNA in the three groups. **(D)** The length of m^6^A-modified circRNAs in the three groups. **(E)** The source of m^6^A-modified circRNAs in the three groups. **(F)** Chromosome distribution.

Further analysis of the MeRIP-seq data was performed, and the m^6^A methylation peaks were compared and analyzed based on the sequencing data. There were 1,599 m^6^A methylation peaks within 1,035 circRNAs in the control group, 35 peaks within 32 circRNAs in the *S. aureus* group, and 1,016 within 728 circRNAs in the *E. coli* group (**Figures 2A, B**).

### Characteristics of m^6^A-Modified circRNAs in MAC-T Cells Treated With Inactivated *S. aureus* and *E. coli*

A total of 17 m^6^A peaks and 18 circRNA transcripts were observed in all three groups, whereby 933 methylation peaks and 687 circRNAs occurred in both the *E. coli* group and the control group. The number of m^6^A methylation peaks was obviously different among the three groups. There were 18 m^6^A peaks in the *S. aureus* group instead of the control group and 83 m^6^A peaks in the *E. coli* group instead of the control group.

Further analysis was performed to assess the features of m^6^A-modified circRNAs. The number of m^6^A methylation peaks in each circRNA was highly similar in the control group, *S. aureus* group, and *E. coli* group (**Figure 2C**). Similar to recent studies, we have found that most circRNAs have one m^6^A methylation peak, but there are also a large number of circRNAs with more than one m^6^A methylation peak, which indicates that m^6^A modification sites are not unique in circRNAs. Moreover, the lengths of m^6^A-modified circRNAs in each group were analyzed. The length of most m^6^A-modified circRNAs was less than 5,000 bp, and similar characteristics of length were shown in all three groups (**Figure 2D**). In our studies, the sources of the circRNA in the three groups were subsequently analyzed. CircRNAs in the control group and *E. coli* group derived from exons were the most abundant. However, in the *S. aureus* group, there was a certain amount of m^6^A-modified circRNA derived from antisense and sense overlapping (**Figure 2E**). Finally, chromosome distribution also revealed that m^6^A-methylated circRNA is more likely to be present on chromosome 3 (**Figure 2F**).

### Comparison of m^6^A-Modified circRNAs in MAC-T Cells Injured by *S. aureus* and *E. coli*

**Figure 3:**
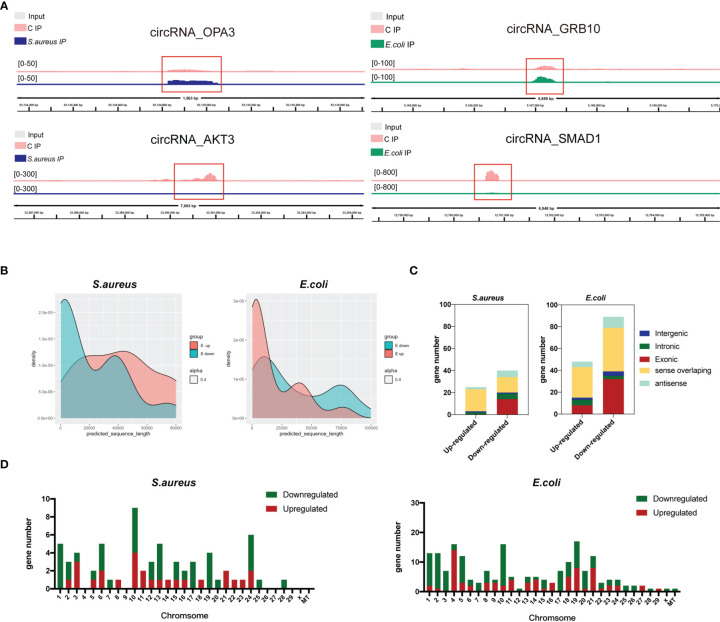
Distribution of significantly differentially expressed m^6^A-modified circRNAs in the *S. aureus/E. coli* group compared to the control group. **(A)** Data visualization analysis was performed by IGV, showing the location of differential m^6^A peaks in the source gene of circRNA (circRNA_OPA3, circRNA_AKT3, circRNA_GRB10, and circRNA_SMAD1) between the control group and treatment group. **(B)** The length, **(C)** source, and **(D)** chromosome distribution of differentially expressed m^6^A-modified circRNAs in the *S. aureus* and *E. coli* groups (circRNAs were named by their host genes).

To identify the role of m^6^A-modified circRNA in regulating MAC-T cell injury induced by *S. aureus* and *E. coli*, the various m^6^A modification levels between *S. aureus/E. coli* and the control group were analyzed (fold change > 2, *p*-value < 0.05). Compared to the control group, a total of 259 differential m^6^A modification peaks within 200 circRNAs were found. Further analysis confirmed that 67 m^6^A methylation peaks within 63 circRNAs were significantly different between the *S. aureus* group and the control group, of which 26 hypermethylated peaks were within 25 circRNAs (e.g., circRNA_OPA3), and 41 hypomethylated peaks were within 38 circRNAs (e.g., circRNA_AKT3). Meanwhile, 192 m^6^A methylation peaks within 137 circRNAs were significantly different between the *E. coli* group and the control group, of which 75 hypermethylated peaks were within 47 circRNAs (e.g., circRNA_GRB10), and 117 hypomethylated peaks were within 90 circRNAs (e.g., circRNA_SMAD1). Data visualization analysis was performed by IGV to show the differential m^6^A peaks between the control group and treatment group (**Figure 3A**). The top 20 differentially methylated circRNAs with hypermethylation or hypomethylation in the *S. aureus* and *E. coli* group compared to the control group are shown (**Tables 1**, **2**).

**Table 1:** The top 20 differential m^6^A methylation peaks in the *S. aureus* group.

Chromosome	txStart	txEnd	circRNA	Log2 (fold change)	Log10 (*p*-value)	Regulation	Gene Name
NC_037337.1	46247281	46247520	NC_037337.1:46242991-46289633+	5.935459748	−5.034591042	Up	HERC1
NC_037330.1	86682301	86682540	NC_037330.1:86640986-86690023-	5.935459748	−5.028395479	Up	FGGY
NC_037333.1	59952041	59952420	NC_037333.1:59928622-59985342-	5.925999419	−5.089972883	Up	APBB2
NC_037350.1	27893981	27894220	NC_037350.1:27876890-27903587+	5.754887502	−5.204708591	Up	BoLA
NC_037332.1	111626261	111626680	NC_037332.1:111598311-111657380+	4.288569498	−5.093922528	Up	TNRC6B
NC_037348.1	67428661	67429080	NC_037348.1:67417009-67430833+	4.161463423	−5.101982705	Up	RCOR1
NC_037348.1	45814301	45814680	NC_037348.1:45804299-45843658-	4.137503524	−5.315886833	Up	RALGAPA1
NC_037337.1	38139881	38140340	NC_037337.1:38110426-38163910-	4.128104826	−5.128618679	Up	TTBK2
NC_037329.1	36254201	36254600	NC_037329.1:36234037-36272150+	4.109121722	−5.205686668	Up	ITGB6
NC_037340.1	17844281	17844540	NC_037340.1:17837769-17914548+	4.109121722	−5.205686668	Up	ABI1
NC_037346.1	48234961	48236200	NC_037346.1:48207986-48238757-	8.727920455	−8.42802011	Down	LOC616254
NC_037346.1	48236381	48236780	NC_037346.1:48207986-48238757-	7.633721813	−5.266786186	Down	LOC616254
NC_037328.1	58661221	58661840	NC_037328.1:58637202-58671946-	7.099084761	−5.035851245	Down	CCDC191
NC_037351.1	41480421	41480700	NC_037351.1:41472840-41512183+	7.087462841	−5.118381385	Down	ANKRD12
NC_037328.1	58659161	58659660	NC_037328.1:58637202-58671946-	6.832890014	−5.107495151	Down	CCDC191
NC_037332.1	47863661	47864140	NC_037332.1:47863491-47900636-	3.850392113	−6.070148314	Down	HMGA2
NC_037333.1	50191621	50192140	NC_037333.1:50182671-50193777+	3.646936522	−6.189481195	Down	PCDH7
NC_037346.1	42688021	42689000	NC_037346.1:42679175-42722558+	3.243082999	−5.067635523	Down	TUBG1
NC_037329.1	103364341	103364580	NC_037329.1:103361756-103403647+	3.068303263	−6.201531963	Down	FN1
NC_037337.1	73166041	73166600	NC_037337.1:73152071-73191463+	2.831439424	−5.695444469	Down	SLC38A6

**Table 2:** The top 20 differential m^6^A methylation peaks in the *E. coli* group.

Chromosome	txStart	txEnd	circRNA	Log2 (fold change)	Log10 (*p*-value)	Regulation	Gene Name
NC_037346.1	48215781	48216000	NC_037346.1:48207657-48222864+	6.264911693	−7.942473705	Up	LOC616254
NC_037341.1	19872121	19872560	NC_037341.1:19868638-19873631+	5.533536841	−6.561672065	Up	\
NC_037336.1	19734241	19734720	NC_037336.1:19722613-19745296+	5.291904302	−6.043718789	Up	TTK
NC_037348.1	21598761	21599100	NC_037348.1:21597478-21609841+	4.240590206	−7.05135328	Up	SEMA4B
NC_037343.1	37622541	37622900	NC_037343.1:37601990-37655462-	3.972129944	−7.178701679	Up	KIFAP3
NC_037333.1	6116441	6116840	NC_037333.1:6115763-6116870-	3.694482263	−6.112188664	Up	MYOZ2
NC_037348.1	45811741	45812060	NC_037348.1:45804299-45843658-	3.651051691	−5.006313952	Up	RALGAPA1
NC_037345.1	64445561	64445840	NC_037345.1:64432982-64467888+	3.491853096	−5.647799044	Up	LOC523461
NC_037341.1	12378481	12378700	NC_037341.1:12358524-12409607-	3.248761682	−6.828578301	Up	\
NC_037351.1	57355041	57355300	NC_037351.1:57288851-57364177+	3.236634344	−6.569723837	Up	NEDD4L
NC_037346.1	42710681	42711440	NC_037346.1:42679175-42722558+	8.250298418	−9.472120196	Down	TUBG1
NC_037332.1	116173961	116174480	NC_037332.1:116173575-116176816+	7.908092341	−8.324295886	Down	\
NC_037341.1	60850021	60850740	NC_037341.1:60844229-60863728-	6.928370323	−5.973128449	Down	RIMS2
NC_037328.1	83737581	83738220	NC_037328.1:83704005-83760741+	6.806066226	−5.801208675	Down	MCF2L2
NC_037346.1	42688021	42689000	NC_037346.1:42679175-42722558+	5.343541908	−7.512392728	Down	TUBG1
NC_037335.1	59571001	59571660	NC_037335.1:59569743-59571921-	4.80910047	−8.534329344	Down	UNC13B
NC_037335.1	61952521	61952820	NC_037335.1:61946441-61961703+	3.936127817	−6.501032761	Down	DCAF10
NC_037334.1	6684921	6685640	NC_037334.1:6684920-6685725+	3.758138793	−5.31671853	Down	KLF2
NC_037337.1	12478301	12478800	NC_037337.1:12457121-12500457-	3.730500053	−9.041917911	Down	INTS14
NC_037344.1	42316401	42316900	NC_037344.1:42315517-42318215+	3.617184214	−9.125043078	Down	PDGFC

Additionally, the feature of the differentially m^6^A-methylated circRNA in the *S. aureus/E. coli* group compared to the control group is also shown. Instead of being a major part of short m^6^A-modified circRNA derived from exon (**Figure 2D**), it is longer and derived more from sense overlapping when MAC-T cells are injured by *S. aureus* and *E. coli* (**Figures 3B, C**). Additionally, the differential circRNA with methylation showed totally reverse patterns in the *S. aureus* group compared to the *E. coli* group, which were mainly hypomethylation in the *S. aureus* group and hypermethylation in the *E. coli* group. The most differentially methylated circRNA in the *S. aureus* group was found on chromosome 10, while in the *E. coli* group, it was on chromosome 19 (**Figure 3D**).

### GO and KEGG Pathway Enrichment Analyses of m^6^A-Modified circRNAs in MAC-T Cells Treated With *S. aureus* and *E. coli*

**Figure 4:**
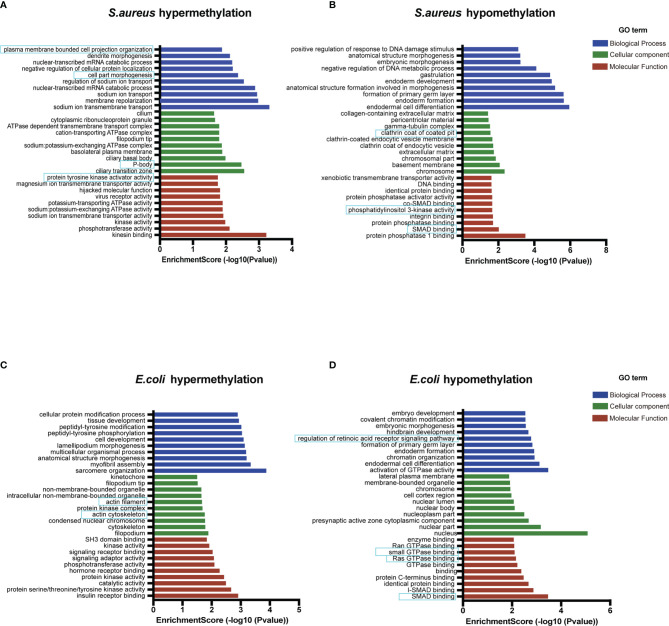
Go enrichment analysis. **(A)** The top 10 GO terms enriched for the source genes of differential circRNA with hypermethylation and **(B)** hypomethylation in the *S. aureus* group. **(C)** The top 10 GO terms enriched for the source genes of differential circRNA with hypermethylation and **(D)** hypomethylation in the *E. coli* group (the predicted GO pathways in the blue box were related to inflammation, apoptosis, and autophagy).

**Figure 5:**
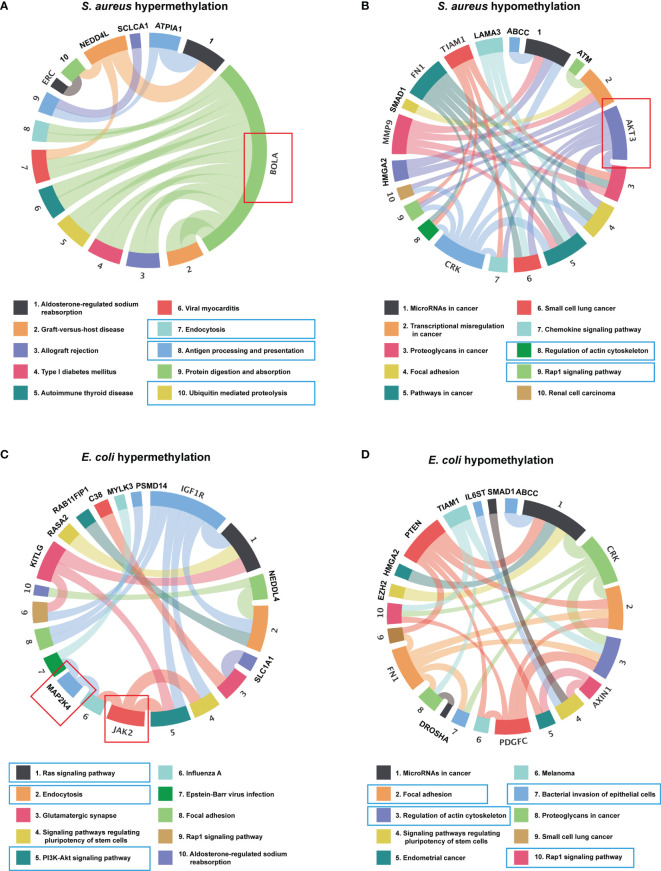
KEGG enrichment analysis. **(A)** The top 10 KEGG pathway enriched for the source genes of differential circRNA with hypermethylation and **(B)** hypomethylation in the *S. aureus* group. **(C)** The top 10 KEGG pathway enriched for the source genes of differential circRNA with hypermethylation and **(D)** hypomethylation in the *E. coli* group (the predicted KEGG pathways in the blue box and representative genes in the red box were related to inflammation, apoptosis, and autophagy).

Recent studies have shown that circRNAs can affect the expression of its cis genes (42). Therefore, to clarify the essential role of m^6^A-modified circRNAs in MAC-T cell injury induced by *S. aureus* and *E. coli*, GO and KEGG pathway enrichment analyses for the m^6^A-modified circRNA source genes were performed (*p*-value < 0.05) (**Tables S3, S4**). In the *S. aureus* group, the GO annotation of circRNA with hypermethylation illustrated that it was mainly enriched in transmembrane, cell part morphogenesis, plasma membrane-bounded cell projection organization, P-body, and protein tyrosine kinase activator activity (**Figure 4A**). Furthermore, the KEGG analyses indicated that they were enriched in endocytosis, antigen processing and presentation, and ubiquitin-mediated proteolysis (**Figure 5A**). Likewise, the GO analysis of circRNA with hypomethylation showed that it was closely related to clathrin, SMAD binding, integrin binding, and PI3K (**Figure 4B**). KEGG analysis of hypomethylated circRNAs revealed enrichment in focal adhesion, the RAP1 signaling pathway, and regulation of the actin cytoskeleton (**Figure 5B**).

In the *E. coli* group, the GO analysis predicted that the functions of circRNAs with hypermethylation were significantly enriched in cytoskeleton, and actin filament (**Figure 4C**). The KEGG pathway analysis indicated that circRNAs with hypermethylation were significantly related to the Ras signaling pathway, endocytosis, and PI3K-Akt signaling pathway (**Figure 5C**). Correspondingly, the GO analysis of differential m^6^A-modified circRNA with hypomethylation illustrated that the meaningful terms may be related to regulation of the retinoic acid receptor signaling pathway, SMAD binding, Ras GTPase binding, and small GTPase binding (**Figure 4D**). The KEGG analysis also revealed enrichment in focal adhesion, regulation of actin cytoskeleton, bacterial invasion of epithelial cells, and Rap1 signaling pathway (**Figure 5D**).

Also, some representative genes closely related to apoptosis and inflammation in the KEGG pathways analysis were shown, such as BOLA, AKT3, MAP2K4, and JAK2 (**Figures 5A–D**). Given the above, we found that the functions of differentially expressed m^6^A-modified circRNAs were highly similar when MAC-T cell injury was induced by either *S. aureus* or *E. coli*. These findings may suggest that these m^6^A-modified circRNAs regulated immunity, cell junctions, growth metabolism, and resistance to bacterial invasion through a similar pathway in both induced groups.

### CircRNA–miRNA–mRNA Interaction Network Identified Similar Enrichment of mRNA in the *S. aureus* and *E. coli* Groups

**Figure 6:**
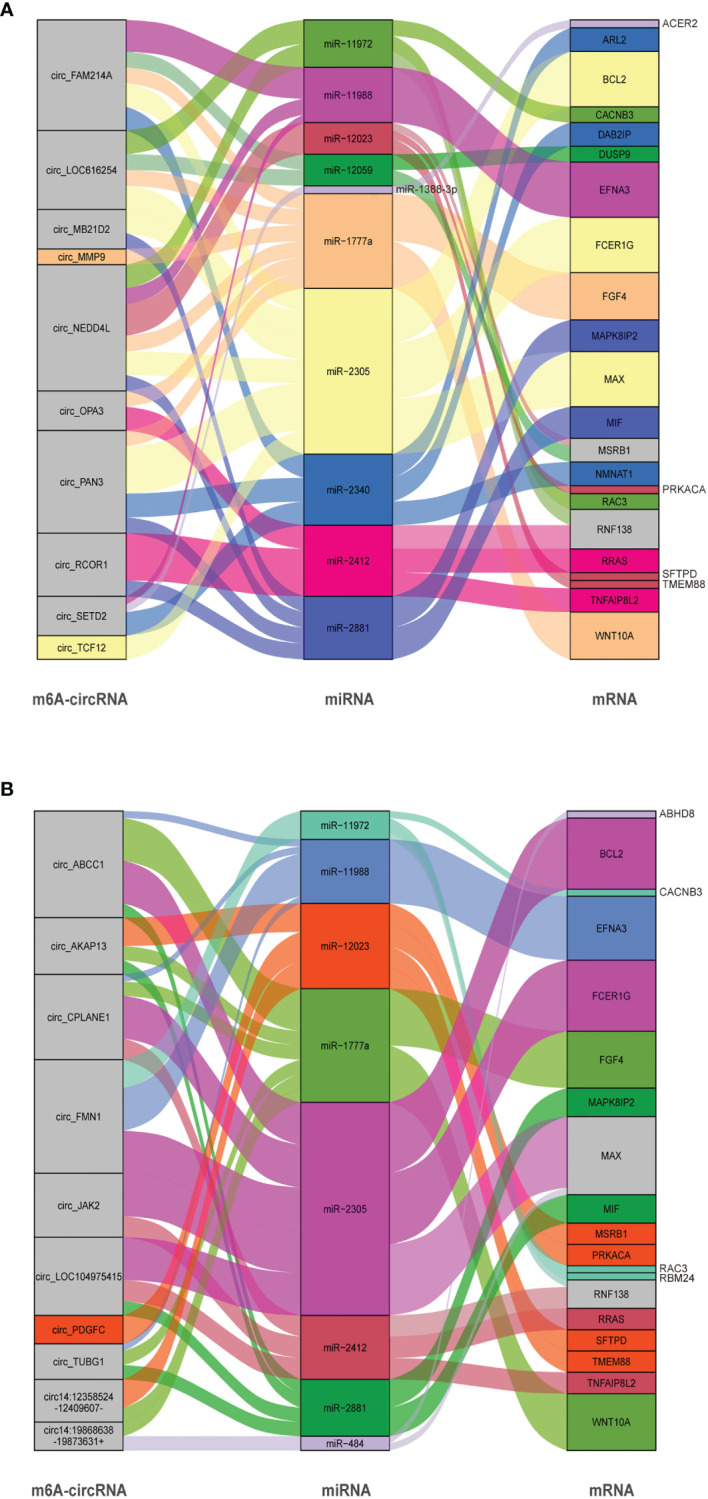
CircRNA–miRNA–mRNA ceRNA interaction network. The box size represents the strength of the binding. The color represents that the miRNA has a binding site with mRNA and circRNA, and if the color is gray, it represents two or more target binding sites. **(A)** The *S. aureus* group. **(B)** The *E. coli* group (circRNAs were named by their host genes or chromosome location).

CircRNAs are able to regulate the expression of target genes as sponges for miRNAs based on complementary base pairing. By predicting the target miRNA between circRNA and mRNA, we constructed a circRNA–miRNA–mRNA interaction network (**Table S5**). In this study, 10 circRNAs (5 hypermethylation and 5 hypomethylation), 10 miRNAs, and 22 mRNAs were included in the *S. aureus* group (**Figure 6A**), and 10 circRNAs (5 hypermethylation and 5 hypomethylation), 8 miRNAs, and 19 mRNAs were included in the *E. coli* group (**Figure 6B**).

In the interactive network of the *S. aureus* group, many inflammation-related miRNAs were predicted. For example, miR-2305 and miR-1777a, which have been considered to have an effect on immunity (43), were also shown to enrich for transcripts related to inflammation and apoptosis in our study (including key genes such as MAPK8IP2 and WNT10A). Additionally, we predicted that some miRNAs may be regulated by multiple circRNAs at the same time. For instance, miR-11988 was predicted to be regulated by circ_FAM214A, circ_NEDD4 L, and circ_SETD2 simultaneously.

Similarly, in the *E. coli* group, a large amount of mRNA was also predicted to be associated with inflammation, mainly in the MAPK signaling pathway and the WNT signaling pathway. In addition, BCL2, which is the key molecule in apoptosis, was predicted to be regulated by miR-2305. Some natural immune-related mRNAs such as TNFAIP8L2, FCER1G, and MSR1B were present in the network. It is predicted that the miRNAs may regulate multiple mRNAs. For example, miR-2305 could be able to interact with BCL2, FCER1G, and MAX. Interestingly, in both groups, we found a large number of similar miRNAs, suggesting that circRNAs modified with different m^6^A modifications may play a similar role in *S. aureus-* and *E. coli-*induced cell injury.

### Conjoint Analysis of circRNA-seq and MeRIP-seq

To further explore the potential function of circRNAs with m^6^A modification in *S. aureus-* and *E. coli-*induced cell injury, a conjoint analysis of circRNA-seq and MeRIP-seq was performed.

**Figure 7:**
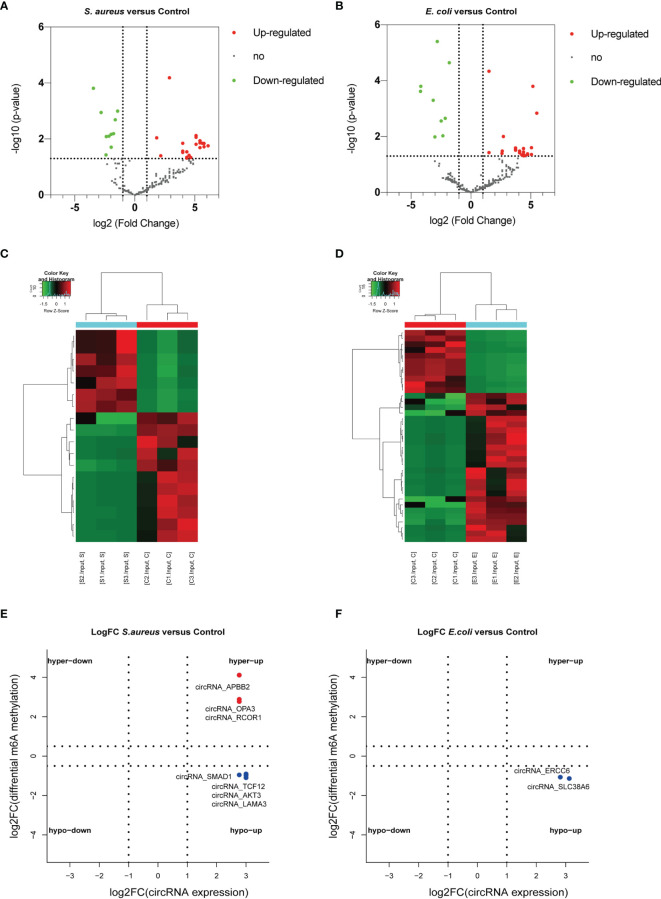
Conjoint analysis of circRNA with or without m^6^A modification. Volcano plot showing the differentially expressed circRNAs in the **(A)** *S. aureus* group and **(B)** *E. coli* group. Heatmap illustrating the differentially expressed circRNAs in the **(C)** *S. aureus* group and **(D)** *E. coli* group. Conjoint analysis between the expression level and m^6^A modification in the **(E)** *S. aureus* group and **(F)** *E. coli* group.

In addition, the differential expression of circRNAs with significant changes in the *S. aureus* and the *E. coli* groups compared to the control group was analyzed (**Figures 7A–D**). We found 3,914 novel circRNAs, of which 1,532 were upregulated and 11 were downregulated in the *S. aureus* group, and 536 were upregulated and 11 were downregulated in the *E. coli* group. The top 20 most significantly differentially expressed circRNAs in the *S. aureus* group and *E. coli* group are listed separately (**Tables S6, S7**).

To predict the especially important circRNA in MAC-T injury and reveal the relationship between m^6^A modification and expression, a conjoint analysis was performed. Finally, 3 significantly upregulated circRNAs with hypermethylation and 4 upregulated circRNAs with hypomethylation were found in the *S. aureus* group (**Figure 7E**). Two upregulated circRNA with hypomethylation were found in the *E. coli* group (**Figure 7F**).

## Discussion

**Figure 8:**
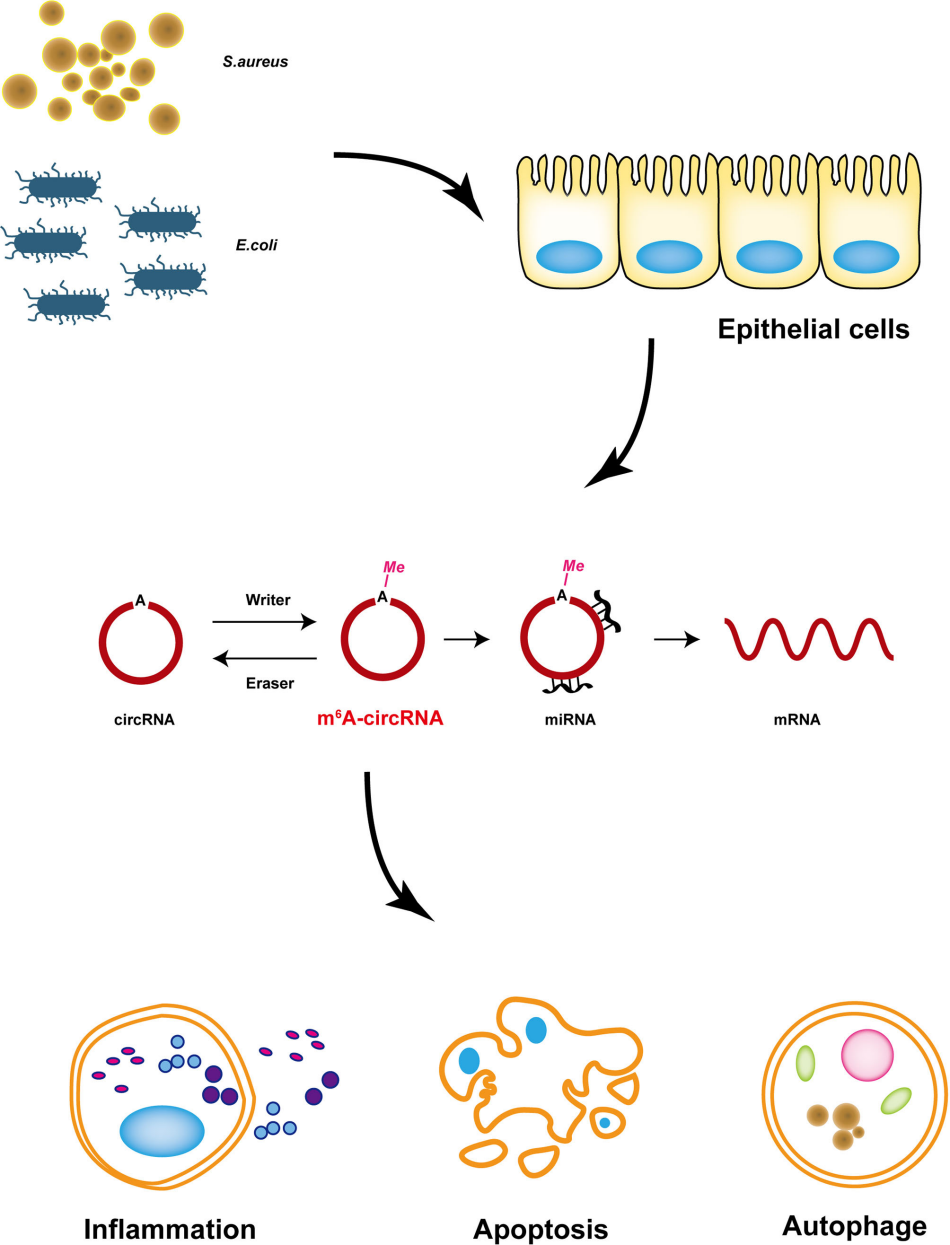
Proposed model of m^6^A-modified circRNAs that regulate MAC-T cells injured by inactivated *S. aureus* and *E. coli.* m^6^A-modified circRNAs regulated MAC-T cell apoptosis, inflammation, and autophagy.

Bovine mastitis is a common disease that seriously harms the dairy industry (44). Currently, antibiotics are still the major treatment for mastitis, which also causes some serious problems, such as drug resistance and threatening side effects. Recent studies have confirmed that circRNA plays an important role in diseases (45), and it can be modified by N^6^-methyladenosine and plays a more complex regulatory role (46). Nevertheless, the mechanism of m^6^A-modified circRNA in bovine mastitis is still undiscovered. This study is the first to reveal the relationship between m^6^A-modified circRNA and cell injury induced by *S. aureus* and *E. coli*, and it reveals that circRNA with m^6^A modification may be related to cell injury by *S. aureus* and *E. coli* treatment (**Figure 8**).

It was reported that cell injury plays an important role in mastitis induced by *S. aureus* and *E. coli* (14). Perhaps the most compelling finding was that IL-1β, IL-6, and TNF-α were significantly upregulated, which indicated inflammatory changes. Additionally, apoptosis and autophagy are common cell injuries during inflammation (47). Inflammatory mRNA expression levels and TEM illustrations both demonstrated that *E. coli* induced more accelerated cell injury.

It is known that m^6^A modification of circRNA usually changes the development of disease. In our research, we found some novel m^6^A peaks first, and also identified the differential m^6^A-modified circRNA profile in the *S. aureus* group and *E. coli* group. This result suggested that this circRNA with different levels of m^6^A modification may play a vital role in *S. aureus-* and *E. coli-*induced cell injury. Most circRNAs upstream of mRNAs are able to regulate gene expression (48), so the altered m^6^A-modified circRNA could be a potential treatment target in mastitis. Furthermore, the unique structure of the conserved covalent closed loop allowed circRNA to remain stable, which makes it a better target than mRNA or other linear RNAs.

CircRNAs as a result of the diverse splicing of mRNAs, and a large number of studies have identified that the major source of circRNAs is derived from exons (49–51), which was also proven in our research. Some researchers have found that circRNAs are produced through contranscription and competition with conventional splicing (52). As a result, biogenesis of circRNA leads to a decrease in mRNA synthesis from the same location. Here, circRNA production acts as an RNA trap for mRNA production. However, we found that the features of m^6^A-modified circRNAs changed when MAC-T cells were injured by *S. aureus* and *E. coli*. Most of the differential m^6^A-modified circRNAs are longer and come from sense overlapping regions, which means that these differential and long m^6^A-modified circRNAs derived from sense overlapping regions play a more important function and provide us with a new direction in the m^6^A-modified circRNA regulatory mechanism of mastitis. In addition, the m^6^A-modified circRNA in both groups showed reverse density patterns of length, which may suggest a difference in cell injury in *S. aureus* and *E. coli* treatment. In fact, there are currently few studies on the m^6^A methylation of circRNA, and whether m^6^A modification can regulate mastitis in dairy cows needs further confirmation.

Since the function of circRNA in dairy cow mastitis is not fully understood, we performed GO/KEGG analysis to predict the function of altered m^6^A methylated circRNA. Previous studies have confirmed that PI3K-AKT and Ras-MAPK are important signaling pathways of natural immunity (53, 54). Li et al. found that ulinastatin reduced LPS-induced inflammation in mouse macrophage cells by activating the PI3K/Akt/Nrf2 pathway (55). Dong et al. found that *Astragalus* polysaccharides alleviate LPS-induced inflammation *via* the NF-κB/MAPK signaling pathway (56). When epithelial cells are injured by bacteria, the tight junctions between cells are compromised through focal adhesion and actin cytoskeletal signaling pathways (57). In our research, we predicted that the differentially expressed circRNAs with m^6^A modification were enriched in the Ras-MAPK, focal adhesion, and PI3K-AKT signaling pathways in MAC-T cells injured by *S. aureus* and *E. coli*, which are closely related to cell apoptosis and inflammation. The pathways and GO annotations of differentially expressed m^6^A-modified circRNAs in the *S. aureus* and *E. coli* groups were highly similar.

Many studies have shown that circRNA can be used as a molecular sponge to interact with miRNA to regulate mRNA (22). Recent studies have proven that specific circRNA–miRNA–mRNA axes cause apoptosis and inflammation in bovine mammary epithelial cells (58). In our ceRNA network, we found that 20 circRNAs and 26 mRNAs had a total of 10 miRNA binding sites, which were obviously related to apoptosis and inflammation. MAX, an important apoptosis gene, was upregulated by direct binding to the miR-181a promoter to increase MYCT1 expression, leading to apoptosis (59). In our study, we found that miR-2305 was able to regulate MAX and BCL2 in both ceRNA networks, and multiple circRNAs had binding sites with miR-2305 (60). In addition, some researchers have shown that downregulation could block the AKT pathway, inhibiting cell survival and proliferation, which means that MIF may be an important gene working in cell proliferation (61).

When MAC-T cells were treated with *S. aureus* and *E. coli*, we found that circ_RCOR1, circ_NEDD4 L, circ_MB21D2, and circ_PAN3 regulate MIF through miR-2881 in the *S. aureus* group, and circ_AKAP13, circ_ABCC1, circ_TUBG1, and circ_LOC104975415 regulate MIF through miR-2881 in the *E. coli* group. This ultimately had a potential effect on MAC-T cell proliferation. RAC3 is an NF-κB coactivator closely related to inflammation, whose expression is regulated by TNF, in addition to being a negative regulator of autophagy (62–64). In our research, miR-11972 was predicted to have binding sites for RAC3. Strangely, the expression of IL-1β, IL-6, and TNFα was different in the cell injury model, while the levels of apoptosis in the two groups were similar (**Figures 1A–F**). Through our ceRNA networks, we conjectured that the m^6^A-circRNA regulated the mRNA enriched in some similar pathways of apoptosis, but there may be more complicated mechanisms that were related to inflammation. Overall, circRNAs with different m^6^A modification levels regulated mRNAs enriched in the MAPK, WNT, and natural immune signaling pathways through circRNA–miRNA–mRNA axes and ultimately affected *S. aureus-* and *E. coli-*induced mastitis. In our research, ceRNA networks showed that mRNAs were enriched in MAPK, WNT, and natural immunology signaling pathways in both the *S. aureus* and *E. coli* groups, which may be the reason for the similar levels of apoptosis.

To further reveal the relationship between circRNA and m^6^A modification, we performed an analysis of circRNA-seq. We found a total of 3,914 novel circRNAs with expression differences. The length and source of altered circRNAs in the *S. aureus* and *E. coli* groups are consistent with the circRNA characteristics of previous studies, and most of them are shorter and exon-derived circRNAs. In the conjoint analysis of MeRIP-seq, we found that a total of 9 circRNAs showed a significant association between expression and m^6^A modification. To change circRNA expression, m^6^A modification has many functions, such as regulated circRNA splicing and degradation (45). For instance, Li et al. confirmed that circMETTL3 is upregulated in a m^6^A-dependent manner and promotes breast cancer progression (65).

This study is the first report to clarify the profile of m^6^A-modified circRNAs in bovine mammary epithelial cells injured by *S. aureus* and *E. coli.* Additionally, we predicted similar pathways and target genes in both groups. This finding provided us with new insights for exploring the cellular injury mechanism of mastitis. However, we did have some limitations. For the purpose of exploring the innate immune response, we used heat-inactivated bacteria instead of live bacteria, which may differ from real mastitis disorders in bovines. In our ceRNA networks, only the circRNAs with different m^6^A levels are shown, without a detailed description of the “up–down–up” or “down–up–down” model of differential expression. Therefore, the mechanism of cell injury needs to be revealed by further experiments, and animal models are needed to clarify the complex function of m^6^A-modified circRNAs *in vivo.*

## Conclusion

Mastitis is a common disease with a complicated pathological mechanism in bovines. m^6^A-modified circRNA plays an important role in regulating disease, but its function in mastitis remains unknown. Our study clearly profiled the m^6^A methylation of circRNA in MAC-T cell injury induced by *S. aureus* and *E. coli* and made predictions on differential m^6^A-methylated circRNA firstly. We provided novel insight into MAC-T cell injury induced by *S. aureus* or *E. coli via* m^6^A-modified circRNAs, which may represent a new research direction for future studies in mastitis.

## Data Availability Statement

The original contributions presented in the study are publicly available. These data can be found here: https://www.ncbi.nlm.nih.gov/geo/query/acc.cgi?acc=GSE196736.

## Author Contributions

HX and CL: Conceptualization, Validation, Formal analysis, Investigation, Resources, Data curation, Writing—original draft, Visualization, Writing—review and editing, and Supervision. YZ and TL: Validation, Formal analysis, and Investigation. JY and SC: Writing—review and editing. CH, JC, XC, YC, and AG: Project administration and Funding acquisition. All authors contributed to the article and approved the submitted version.

## Funding

This research was funded by the National Natural Science Foundation of China (No. 31972758; 31101874).

## Conflict of Interest

The authors declare that the research was conducted in the absence of any commercial or financial relationships that could be construed as a potential conflict of interest.

## Publisher’s Note

All claims expressed in this article are solely those of the authors and do not necessarily represent those of their affiliated organizations, or those of the publisher, the editors and the reviewers. Any product that may be evaluated in this article, or claim that may be made by its manufacturer, is not guaranteed or endorsed by the publisher.

## Supplementary Material

Supplementary Figure 1 Overview of the circRNA profile. **(A)** Novel circRNAs, mainly exonic, intronic, intergenic, sense overlapping, and antisense circRNAs. **(B)** The length of the novel circRNA found by circRNA-seq. **(C, D)** Pie charts showing the length of differentially expressed circRNAs in the *S. aureus* and *E. coli* groups. **(E)** The chromosome distribution of the novel circRNAs. **(F) **Histogram showing the source of differentially expressed circRNAs in the *S. aureus* and *E. coli* groups. **(G)** The chromosome distribution of differential circRNAs in the *S. aureus* and *E. coli* groups.
