# Supplementary material for: N6-Methyladenosine-Modified circRNA in the Bovine Mammary Epithelial Cells Injured by Staphylococcus aureus and Escherichia coli
Source: Front Immunol. 2022 Apr 4;13:873330. doi: 10.3389/fimmu.2022.873330 (PMC9014013; doi:10.3389/fimmu.2022.873330)
Supplement: Supplementary Figure 1 — Overview of the circRNA profile. (A) Novel circRNAs, mainly exonic, intronic, intergenic, sense overlapping, and antisense circRNAs. (B) The length of the novel circRNA found by circRNA-seq. (C, D) Pie charts showing the length of differentially expressed circRNAs in the S. aureus and E. coli groups. (E) The chromosome distribution of the novel circRNAs. (F) Histogram showing the source of differentially expressed circRNAs in the S. aureus and E. coli groups. (G) The chromosome distribution of differential circRNAs in the S. aureus and E. coli groups. [file DataSheet_1.zip › Supplementary Tables and Figure/Table S7 The top 20 differentially expressed circRNA in E.coli group..docx]

**Table S7. The top 20 differentially expressed circRNA in *E. coli* group**

| **circRNA ID** | **Gene Name** | **Log2 (Fold Change)** | **Log10 (*p*-Value)** | **Regulation** |
| --- | --- | --- | --- | --- |
| NC_037334.1:50134386-50146696+ | CTNNA1 | 5.502572155 | -2.839149532 | up |
| NC_037328.1:69271497-69301725- | ITGB5 | 5.169238825 | -3.797710452 | up |
| NC_037342.1:6545342-6569679- | YAP1 | 5.061929456 | -1.35817589 | up |
| NC_037332.1:44567382-44580289- | CPSF6 | 5.061929456 | -1.604211011 | up |
| NC_037338.1:40722964-40728475+ | VRK2 | 4.728596125 | -1.37583847 | up |
| NC_037339.1:16089515-16106617- | ZC3H13 | 4.728596125 | -1.37583847 | up |
| NC_037340.1:15989889-15999305- | TAF3 | 4.728596125 | -1.37583847 | up |
| NC_037332.1:29476298-29481624- | LARP4 | 4.728596125 | -1.37583847 | up |
| NC_037355.1:24832129-24839488- | DNA2 | 4.728596125 | -1.380765783 | up |
| NC_037348.1:5300954-5301449+ | CHSY1 | 4.728596125 | -1.35556783 | up |
| NC_037337.1:67199994-67200512+ | SAMD4A | -4.207841482 | -3.615733807 | down |
| NC_037348.1:57913323-57915074- | BTBD7 | -4.184378375 | -3.795421547 | down |
| NC_037329.1:43867559-43898911- | / | -3.141284034 | -3.295278836 | down |
| NC_037341.1:12458977-12543218- | TRNAG-CCC | -3.002938192 | -1.985242472 | down |
| NC_037333.1:17503709-17523590+ | PAPSS1 | -2.807950694 | -5.402431348 | down |
| NC_037329.1:43596900-43602712- | FMNL2 | -2.474617358 | -2.558302005 | down |
| NC_037329.1:43892092-43892471- | / | -2.336271532 | -2.029831215 | down |
| NC_037330.1:98890783-98896599- | CMPK1 | -2.336271532 | -2.025266454 | down |
| NC_037345.1:54183330-54204953+ | SAE1 | -2.141284034 | -2.651279501 | down |
| NC_037350.1:3019707-3035565+ | RAB23 | -1.807950715 | -4.640932272 | down |
